# OM-VST: A video action recognition model based on optimized downsampling module combined with multi-scale feature fusion

**DOI:** 10.1371/journal.pone.0318884

**Published:** 2025-03-06

**Authors:** Xiaozhong Geng, Cheng Chen, Ping Yu, Baijin Liu, Weixin Hu, Qipeng Liang, Xintong Zhang

**Affiliations:** 1 Changchun Institute of Technology, Changchun, Jilin, China; 2 Jilin Institute of Chemical Technology, Jilin, Jilin, China; 3 Jilin University of Finance and Economics, Changchun, Jilin, China; Bu-Ali Sina University: Bu Ali Sina University, IRAN, ISLAMIC REPUBLIC OF

## Abstract

Video classification, as an essential task in computer vision, aims to identify and label video content using computer technology automatically. However, the current mainstream video classification models face two significant challenges in practical applications: first, the classification accuracy is not high, which is mainly attributed to the complexity and diversity of video data, including factors such as subtle differences between different categories, background interference, and illumination variations; and second, the number of model training parameters is too high resulting in longer training time and increased energy consumption. To solve these problems, we propose the OM-Video Swin Transformer (OM-VST) model. This model adds a multi-scale feature fusion module with an optimized downsampling module based on a Video Swin Transformer (VST) to improve the model’s ability to perceive and characterize feature information. To verify the performance of the OM-VST model, we conducted comparison experiments between it and mainstream video classification models, such as VST, SlowFast, and TSM, on a public dataset. The results show that the accuracy of the OM-VST model is improved by 2.81*%* while the number of parameters is reduced by 54.7*%*. This improvement significantly enhances the model’s accuracy in video classification tasks and effectively reduces the number of parameters during model training.

## 1 Introduction

Convolutional neural networks (CNNs) are the classical approach in computer vision tasks [1]. CNNs can capture local feature information and perform feature extraction through parameter sharing, effectively reducing the network model’s computational complexity and improving computational efficiency. Additionally, by directly inputting image data into the network, CNNs avoid the extra step of manual feature extraction [2–4]. However, CNN only focuses on local feature extraction, which makes it difficult to perceive global information effectively, and the computational resource demand increases dramatically for deep network structures. These defects hinder the development of CNN to some extent.

The Google team first introduced an image classification network based on the Transformer architecture named Vision Transformer (ViT) [5] to address the limitation of CNN models in extracting global information. ViT divides the input image into 16×16 patches, each containing partial details on the feature map. This enables the network to extract local features effectively, allowing for accurate recognition of objects that are widely distributed and numerous within the image. The ViT network successfully overcomes the issue of CNN’s weak ability to capture long-range pixel relationships, allowing it to capture richer feature information and significantly improving model classification performance [6]. However, the self-attention mechanism of ViT requires substantial computational resources. For deeper networks or high-resolution images, the computational demands of the ViT architecture are even more significant.

To solve the problem of ample resources required for Transformer network computation, researchers at Microsoft Research Asia designed a hierarchical Transformer model named Swin Transformer [7]. Swin Transformer refers to the idea of image modularization for the ViT model. It innovatively adopts a Shifted windows design, which enables the model to learn feature information across windows. In addition, the hierarchical model can handle super-resolution image information, which saves the computational resources required for model training. Swin Transformer has higher scalability and fewer computational resource requirements than ViT. However, the Swin Transformer is not computationally efficient and is not sufficiently aware of complex feature information.

We propose an improved model based on the Video Swin Transformer (VST) to address the above problems. To improve the computational efficiency of the model, with the ability to perceive complex feature information, we improve the VST model in two aspects. In the optimized downsampling module of the model, we increase the size of the convolutional kernel and refer to the Inverted Bottleneck architecture to adjust the dimensional transformation of the feature map in the downsampling stage, aiming to increase the receptive field and improve the convergence speed of the model. In the feature extraction stage of the model, we add a multi-scale information fusion module to capture complex feature information by weighting the feature information of two different convolutional branches to improve the computational efficiency of the model. Through the above improvements, the model can extract richer feature information, which improves the model performance and accelerates the convergence speed of the network at the same time. We have experimentally verified that removing some redundant network layers can effectively enhance the computational efficiency of the model. These improvements enable the model to handle more complex video classification tasks and to show more accurate results with more robust performance in practical applications.

The key contributions of this paper are as follows:

1. We designed the Optimized Downsampling module. This module uses a larger convolutional kernel to increase the receptive field and adjusts the dimension transformation of the feature map in the downsampling stage. This method increases the model receptive field and improves the convergence speed
2. We add a multi-scale feature fusion module in the feature extraction stage further to enhance the feature extraction capability of the model and to speed up the computational efficiency of the model.
3. We appropriately remove the redundant network layers and reduce the model depth, which effectively reduces the number of parameters and accelerates the computational efficiency of the model.
4. Experiments show that our OM-VST model has better video classification performance.

The rest of this paper is organized as follows: Sect 2 reviews correlation theory. Sect 3 introduces our proposed video classification model. Sect 4 presents the experimental results. Sect 5 provides a discussion. Sect 6 provides a conclusion.

## 2 Correlation theory

### C3D network

**Fig 1:**
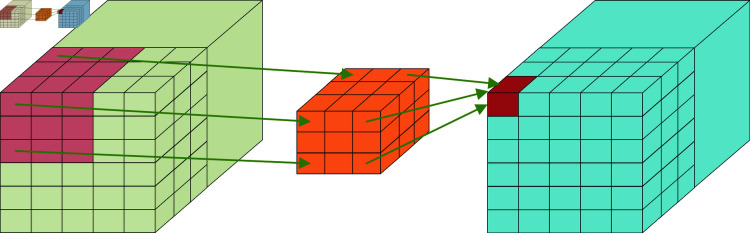
3D convolution.

3D convolutional neural network (C3D), developed by Tran et al. [8]. In 2015, it strengthened the ability of motion modeling based on 2D convolutional neural networks. It has been widely applied to video information processing networks [9]. C3D is a convolution on three channels. Compared with 2D convolution, the time dimension is added [10–12], and the feature information of both time and space can be extracted at the same time [13]. Fig 1 shows 3D convolution. In Fig 1, the filter can move in the direction of the three-dimensional input information’s length, width, and height. At each position, the filter individually integrates the data with the 3D feature map. As the filter slides, the information of the entire input layer is spread, and the final calculated result parameters form a 3D spatial distribution matrix.

### Self-attention mechanism

**Fig 2:**
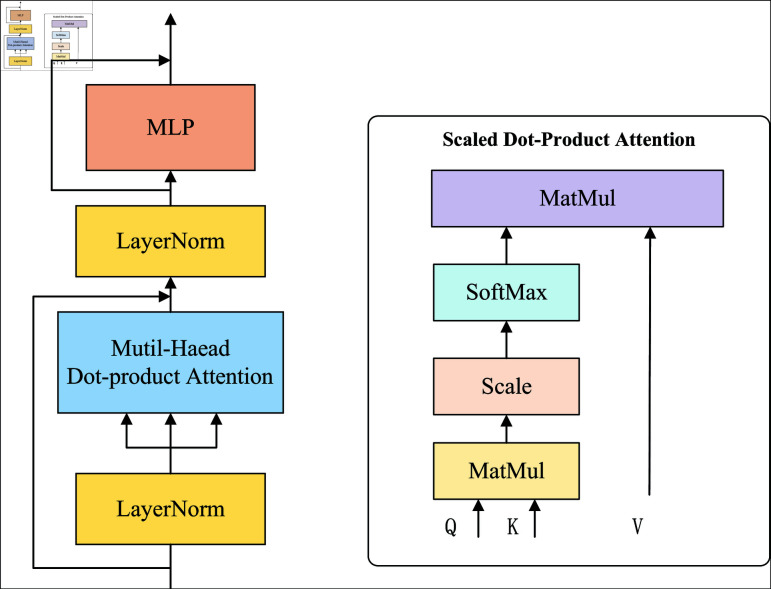
Self-attention module.

The self-attention mechanism in deep learning, also known as internal attention, is commonly used in NLP. In recent years, more and more image tasks have adopted self-attention mechanisms and achieved good results. The self-attention mechanism uses the dot product method to compute the attention weight of each feature map semantic patch using query vector, key vector, and value vector [14,15]. Fig 2 shows the self-attention module, which can improve the model’s ability to focus on essential parts of incoming data and establish connections between distant feature information in the feature map. The self-attention mechanism mainly focuses on the internal feature information interaction of data or feature sequences without the involvement of external information.

The self-attention mechanism encodes pixel-level information of all incoming data. Then, it focuses on the relationships between their feature sequence information to establish a network that correlates global information [16]. The model of this mechanism can learn the information between various elements without requiring positional information in the sequence. The input matrix is used to calculate vectors $q_{\left(p,t\right)}^{\left(\ell ,a\right)}$, $k_{\left(p,t\right)}^{\left(\ell ,a\right)}$ and $v_{\left(p,t\right)}^{\left(\ell ,a\right)}$through three different learnable weight matrices $W_{Q}^{\left(\ell ,a\right)}$, $W_{K}^{\left(\ell ,a\right)}$, $W_{V}^{\left(\ell ,a\right)}$ [17].


$$\begin{array}{ccc} & q_{\left(p,t\right)}^{\left(\ell ,a\right)}=W_{Q}^{\left(\ell ,a\right)}LN\left(z_{\left(p,t\right)}^{\ell -1}\right)\in R^{D_{h}}, & \end{array}$$ (1)

$$\begin{array}{cc} & k_{\left(p,t\right)}^{\left(\ell ,a\right)}=W_{K}^{\left(\ell ,a\right)}LN\left(z_{\left(p,t\right)}^{\ell -1}\right)\in R^{D_{h}}, \end{array}$$ (2)

$$\begin{array}{cc} & v_{\left(p,t\right)}^{\left(\ell ,a\right)}=W_{V}^{\left(\ell ,a\right)}LN\left(z_{\left(p,t\right)}^{\ell -1}\right)\in R^{D_{h}}, \end{array}$$ (3)

In which, *ℓ* = 1, 2,..., L, L represents the total quantity of attention layers, *LN* represents the layer normalization function Layernorm, and z represents the output feature map of the previous model. *D* represents the hidden dimension of the input sequence. a = 1, 2,..., a is the quantity of multi-head attention heads [18], and the dimensionality of the feature map processed by each attention head is $D_{h}=D∕A$.


$$\begin{array}{ccc} Attention(q_{\left(p,t\right)}^{\left(\ell ,a\right)},k_{\left(p,t\right)}^{\left(\ell ,a\right)},v_{\left(p,t\right)}^{\left(\ell ,a\right)})=SoftMax(q_{\left(p,t\right)}^{\left(\ell ,a\right)}{\left(k_{\left(p,t\right)}^{\left(\ell ,a\right)}\right)}^{T}∕\sqrt{d}+B)v_{\left(p,t\right)}^{\left(\ell ,a\right)}, & & \end{array}$$ (4)

By calculating the linear variation of different attention weights, the self-attention values $q_{\left(p,t\right)}^{\left(\ell ,a\right)}$, $k_{\left(p,t\right)}^{\left(\ell ,a\right)}$, $v_{\left(p,t\right)}^{\left(\ell ,a\right)}$ are obtained, which are the query matrix, key matrix, and value matrix, respectively. These matrices determine the weight distribution during parameter training. *B* represents the relative positional deviation. *d* represents the dimension of the query and key features. Divide each element by $\sqrt{d}$ in the above equation to keep the parameter gradient stable during self-attention calculation.

### Transformer

**Fig 3:**
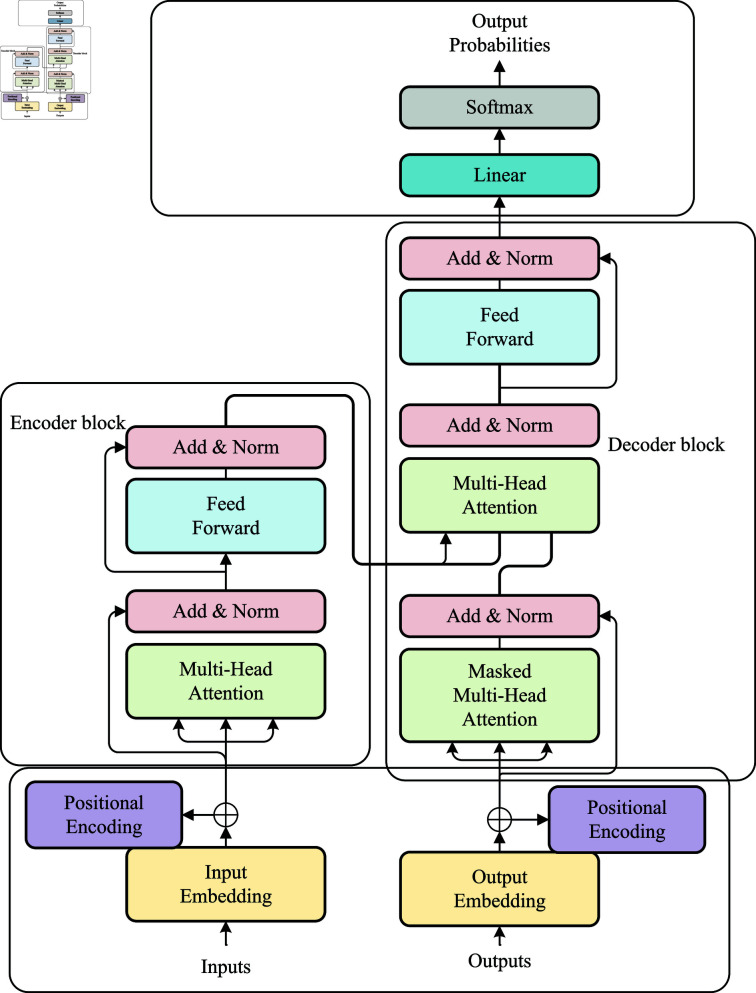
Transformer model network structure.

Transformer is a deep learning model primarily used for NLP tasks, especially Sequence-to-Sequence learning problems such as text generation. The union of the Transformer and self-attention mechanism is a key innovation [19], which makes it outstanding in processing sequential data. The structure of the Transformer is shown in Fig 3.

Transformer’s network structure mainly consists of an encoder and a decoder. The encoder includes a multi-head attention layer and a Feed Forward Network (FFN) layer, which perform multi-head parallel operations on input parameters to learn different attention weights and better capture relationships of different types [20]. FFN includes two linear layers and a ReLU activation function. These two-layer networks map the input feature vectors to a higher dimensional space, filter them through non-linear ReLU, and transform them into their original dimensions [21].

The decoder consists of two multi-head attention layers. The first layer uses a masked operation, and the input of the second layer consists of the output of the previous layer’s multi-head attention layer and the encoded information matrix output of the encoder. The calculation mechanism of the two-layer multi-head attention layer is the same. The previous layer added a masked operation to mask specific parameter values to avoid affecting the results during parameter updates.

### Video swin transformer

**Fig 4:**
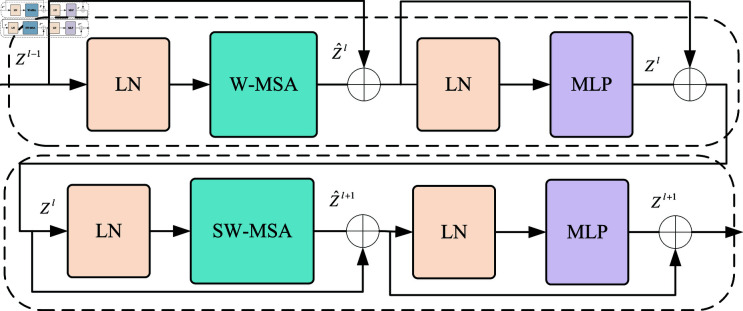
VST Block network structure.

VST network is a hierarchical architecture where the feature map size of the input video information gradually decreases after passing through each level. This structural network can learn multi-scale feature information while accelerating training efficiency [22]. The key innovation of the network is the sliding window design, as shown in Fig 4, which reduces the consumption of computing resources and more effectively learns the global attention feature sequence information [23]. VST can effectively learn the connections between pixels at similar spatiotemporal distances by integrating spatial locality induction bias, hierarchical and translational invariance, and a complete spatiotemporal attention mechanism, thereby enhancing model performance and improving training efficiency [24]. VST network includes a Windows Multi-head Self-attention (W-MSA) module and a Shifted Window Multi-head Self-attention (SW-MSA) module. The input feature map is extracted locally in the window through W-MSA and then entered into SW-MSA for information exchange between windows. SW-MSA first segments and reorganizes the feature map window perform the self-attention operation on the shifted window and finally reorganizes and restores the window. The feature map is finally output by LayerNorm and Multilayer Perception (MLP).

The computational complexity of dense pixel self-attention computation is very high, but using windowed segmentation feature map design can significantly reduce it. After traversing the VST Block network, the input feature map is calculated as follows [7].


$$\begin{array}{ccc} & {ẑ}^{l}=3DW-MSA\left(LN\left(z^{l-1}\right)\right)+z^{l-1} & \end{array}$$ (5)

$$\begin{array}{cc} & z^{l}=MLP(LN({ẑ}^{l}))+z^{l} \end{array}$$ (6)

$$\begin{array}{cc} & {ẑ}^{l+1}=3DSW-MSA(LN(z^{l}))+z^{l} \end{array}$$ (7)

$$\begin{array}{cc} & z^{l+1}=MLP(LN({ẑ}^{l+1}))+z^{l+1} \end{array}$$ (8)

$z^{l}$ and $z^{l+1}$ are for the output features of the 3DW-MSA and 3DSW-MSA, $z^{l}$ and $z^{l+1}$ are for the output features of the MLP module, and 3DW-MSA and 3DSW-MSA are the regular window self-attention and sliding window self-attention modules, respectively.

## 3 Proposed deep learning model

### OM-VST model network structure

**Fig 5:**
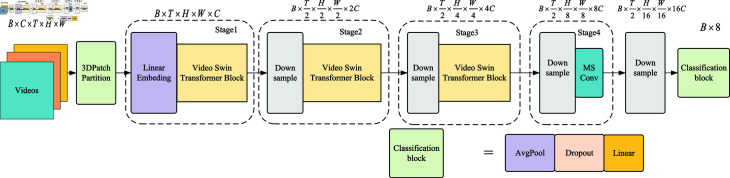
OM-VST model network structure.

The overall network structure of the OM-VST model is similar to the Video Swin Transformer (VST) model, as shown in Fig 5. The model is a hierarchical design that divides the backbone network into four stages. Each stage has a different size of feature maps, and the optimized downsampling module adjusts the size of the feature maps and the number of channels. The feature extraction module extracts information on feature maps of different scales [25].

The optimized downsampling module we propose is divided into two main parts: firstly, the size of the feature map and the number of channels are adjusted by Patch Merging. Unlike traditional downsampling, Patch Merging uses a small number of parameters because it only realizes the change of feature map size by splicing adjacent pixel points, which does not involve calculating parameters. Then, after Patch Merging, we add the feature extraction module. The feature extraction module adopts the idea of large kernel convolution to transform the number of feature map channels. Large kernel convolution is larger than the standard convolution kernel, which plays well in feature extraction after the experiments of Ding et al. In the first few layers of the network, the large kernel convolution is first used to increase the number of channels, which helps the network to extract more feature information. Then, decreasing the number of channels is performed, which helps compress and extract more effective feature information, thus obtaining a more compact feature representation and reducing the number of computational parameters. This module allows the network to get a wider sensory field and deeper semantic information and speeds up the convergence of the model.

We propose a multi-scale feature fusion module to improve the model’s classification performance by fusing information from multiple scales. In this study, we realize the fusion of multi-scale feature information by fusing two different scales of convolutional feature information with weight summation. This module aims to combine various levels of macro and micro information, with large-scale convolution focusing on the wide-area information of the feature map and small-scale convolution focusing more on the detailed performance of the feature map. By fusing these two types of information, richer local features are captured while maintaining the complete shape of the object in the feature map.

We propose that these modules effectively improve the model’s classification performance and significantly reduce the number of covariates computed by the model through different treatments of the feature map.

### Optimized downsampling module

**Fig 6:**
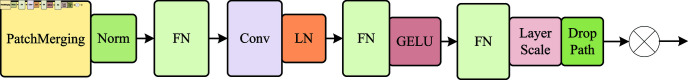
Optimized Downsampling network structure.

Fig 6 shows the network architecture of the optimized downsampling module, which shortens the size of the feature maps while improving the number of feature maps. This structure conforms to the Inverted Bottleneck architecture and the information channel design of “wide in the middle and small at both ends,” which can reduce information loss during network training and retain more effective information [26].

**Fig 7:**
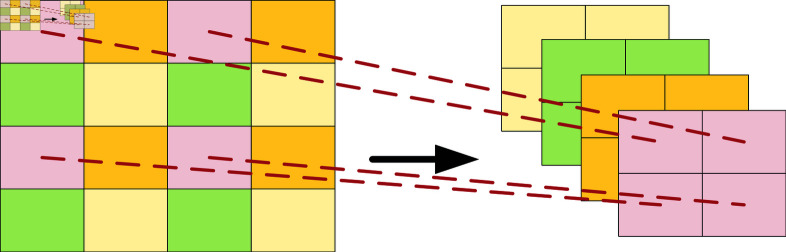
Patch merging network structure.

The patch merging layer in the model, as shown in Fig 7, is used for downsampling to shorten the size of feature maps and adjust the number of channels. It conforms to the hierarchical design of the model and reduces computational complexity.

This model uses less normalization, which can avoid introducing prior knowledge and bias as much as possible while reducing the network’s training time and computational resources. Normalization uses the commonly used Layer Normalization (LN) in Transformer, which can normalize the hidden layer dimensions of all samples, reduce the differences between samples, and make the distribution of samples more stable. It also helps to facilitate the information transmission between different levels in the model learning process, reducing the differences in sample information transmission between different levels.

The convolutional layer design of the optimized downsampling module proposed in this paper conforms to the idea of large kernel convolution [27]. The convolution kernel’s size directly limits the receptive field’s size. Due to its larger kernel size, large kernel convolution can cover more feature map information in one convolution operation, thereby obtaining more upper and lower-layer information and extracting global features.

### Multi-scale feature information fusion module

**Fig 8:**
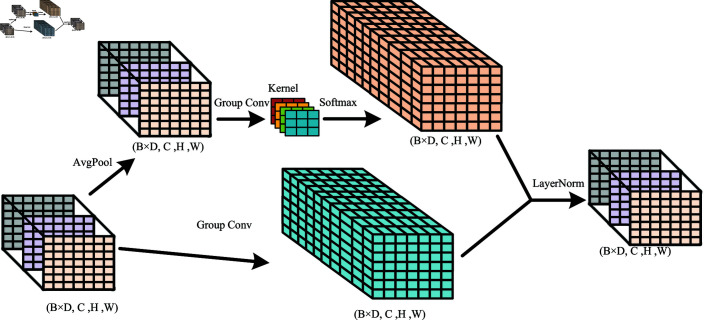
Multi-scale feature information fusion module network structure.

OM-VST model applies a multi-scale feature information fusion module in Stage 4. The structure of the multi-scale feature information fusion module is shown in Fig 8. The module is designed to solve the multi-scale problem in object detection and increase the model’s performance when recognizing objects of different sizes [28]. It enhances the classification performance and robustness of the model by integrating feature information from diverse levels of the network. In the deep levels of the network, the model can obtain a broader range of contextual relationships, thereby extracting global semantic information. At shallower levels, more local details can be obtained. Combining global semantics with local feature information enables the network to understand image content better. In addition, the multi-scale convolution module effectively reduces the loss of feature information. With the increased number of layers in the network, reducing feature map size often leads to information loss, particularly for small targets and detailed features. The multi-scale feature fusion strategy ensures the preservation of feature information at each level in the hierarchical network, increasing the capacity to detect small objects and capture details.

## 4 Results

A series of experiments will verify the feasibility and effectiveness of the OM-VST model. The experiment will be conducted under the PyTorch deep learning framework, using Python version 3.8 and CUDA version 11.3 environments [29,30]. Regarding hardware configuration, an NVIDIA RTX 4090 graphics card will be used, equipped with 24GB of video memory to ensure efficient operation.

The experimental steps are as bellow:

1. Data preprocessing: We collect public human movement video datasets. The videos in the data set are standardized and enhanced to increase the model’s generalization ability.
2. Model training: OM-VST model is used for training on the preprocessed data set, and network parameters are adjusted until convergence.
3. Model evaluation: Evaluate the trained model on the test set and calculate the above evaluation indicators.
4. Comparative experiment: The performance of the OM-VST model is contrasted with that of the traditional model to show its advantages.
5. Ablation experiment: This is used to assess the importance and role of each component of the model.

### Datasets and evaluation indicators

The experimental dataset used in this paper is based on the publicly available video dataset Kinetics400. We extracted eight typical action video categories from the Kinetics400 dataset as the dataset for this experiment. The eight categories include complex human action postures, and there are also similar actions between the categories to verify that the model can recognize subtle gaps between actions. For each action category, the training set consists of 300 videos at 30fps, and the test set consists of 40 videos at 30fps, each of which is approximately 10 seconds long, all saved in MP4 format. Our dataset consists of 8 categories, and a mapping table is created for each category using the numbers 1-8. The mapping is as follows:  < 1: bobbling > ,  < 2: scrolling > ,  < 3: bundling jumping > ,  < 4: capoeira > ,  < 5: catchingfish > ,  < 6: clean_1and_jerk4 > ,  < 7: eating_ice_cream > ,  < 8: triple_jump > .

Table 1 shows the basic parameter settings for our subsequent experiments, and all subsequent experiments will be conducted according to the parameter settings in the table [31].

**Table 1:** Experiment parameter configuration.

*BatchSize*1	epoch	clip_len	frame_interval	Resize
4	200	32	3	224×224

We will describe the evaluation metrics used in this study. The evaluation metrics we used are Accuracy, Precision, Recall, F1-Score, False Positive Rate (FPR), and True Positive Rate (TPR) [32,33].

Accuracy: the proportion of correctly categorized samples to all tested samples.


$$\begin{array}{ccc} Accuracy=\frac{TP+TN}{TP+TN+FP+FN} & & \end{array}$$ (9)

Precision: the proportion of correctly classified positive samples to the proportion of samples classified as positive.


$$\begin{array}{ccc} Precision=\frac{TP}{TP+FP} & & \end{array}$$ (10)

Recall: the proportion of correctly categorized positive samples to all positive samples.


$$\begin{array}{ccc} Recall=\frac{TP}{TP+FN} & & \end{array}$$ (11)

F1-Measure: the reconciled mean of Precision and Recall.


$$\begin{array}{ccc} F1-Measure=\frac{(a^{2}+1)*P*R}{a^{2}*(P+R)} & & \end{array}$$ (12)

FPR: the proportion of misclassified positive samples to all positive samples.


$$\begin{array}{ccc} FPR=\frac{FP}{TN+FP} & & \end{array}$$ (13)

TPR: the proportion of negative samples classified correctly to all negative samples.


$$\begin{array}{ccc} TPR=\frac{TP}{TP+FN} & & \end{array}$$ (14)

TP : True cases, which are predicted to be positive and are actually positive; FP : False positives, which are predicted to be positive but are actually negative; FN : False Negatives, which are predicted to be negative but are actually positive; TN : True Negatives, which are predicted to be negative but are actually negative.

### Result and analysis

Table 2 shows the performance of OM-VST in the public dataset, and the central evaluation metrics include Precision, Recall, and F1-Score. From the results, the model performs very well in all categories, especially in the categories of L2 (bowling) and L8 (triple jump). The F1-Score of classification is more than 90*%*, indicating that the network can fully extract the critical feature information in the video to obtain more accurate classification data. More than 90*%*, indicating that the network can fully extract the critical feature information in the video and thus obtain more precise classification data. Even in the L4 (capoeira) category, which is a poor performer, the F1-Score value reaches 76*%*, up to the current network’s average classification standard. Overall, OM-VST demonstrates a strong generalization ability and can efficiently and accurately classify video content in various complex scenarios.

**Table 2:** Performance comparison of various categories.

*Class*	Precision	Recall	F1-Score
$L_{1}$	0.77	0.90	0.83
$L_{2}$	0.90	0.93	0.91
$L_{3}$	0.91	0.80	0.85
$L_{4}$	0.82	0.70	0.76
$L_{5}$	0.88	0.90	0.89
$L_{6}$	0.82	0.93	0.87
$L_{7}$	0.92	0.85	0.88
$L_{8}$	0.97	0.97	0.97

**Fig 9:**
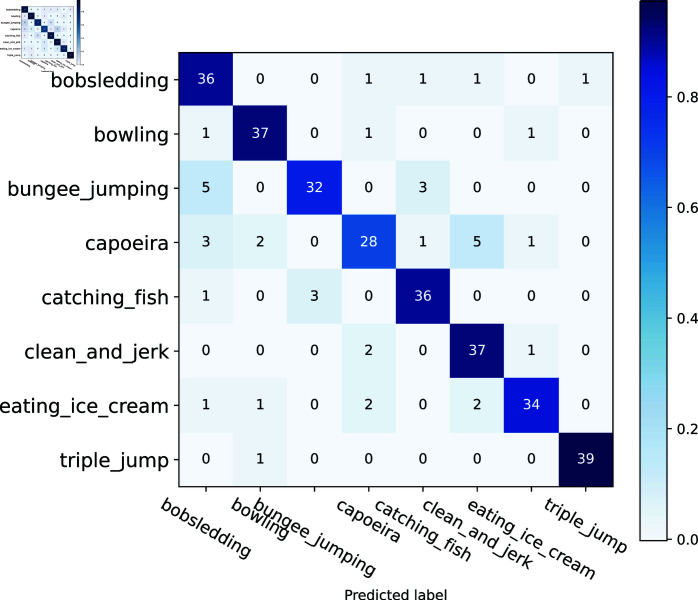
Confusion matrix.

We evaluate the model’s performance by analyzing the Confusion Matrix. The Confusion Matrix can visualize the model’s classification in each category [34]. As shown in Fig 9, 40 samples for each category participated in the experiment, and 6 had more than 34 correct classifications, while only two categories had relatively low classification accuracy. However, overall, the model exhibits high classification performance. Overall, the model exhibits high classification performance. The model is more generalized and performs well in complex multi-category environments, exhibiting strong robustness and high accuracy.

**Fig 10:**
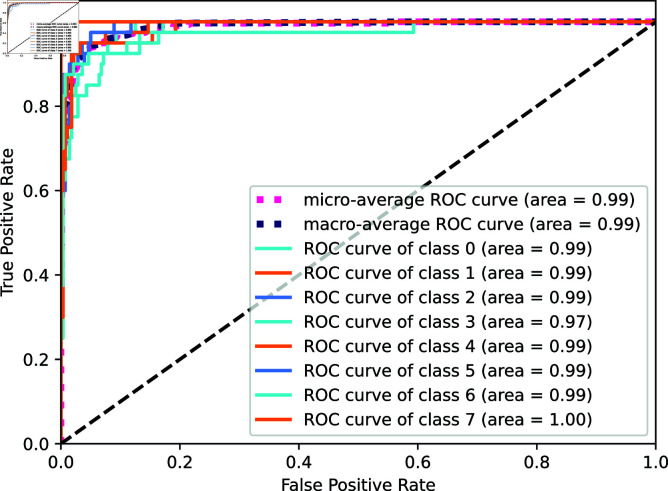
ROC curves.

We evaluate the classification performance of the OM-VST network on a complex dataset by analyzing the ROC curve (Receiver Operating Characteristic Curve) for each category. This dataset combines multiple complexities, including the imbalance between categories and high feature diversity, which makes the classification task more challenging. The horizontal axis of the ROC curve represents the false positive rate (FPR), and the vertical axis represents the actual rate (TPR), which measures the proportion of false positives and true positives in the model’s classification, respectively. As in Fig 10, we show the ROC curves for each category and the micro-averaged and macro-averaged ROC curves. The micro-averaged ROC curves combine the performance of all categories and reflect the overall classification effect.In contrast, the macro-averaged ROC curves focus on the individual performance of each category by averaging the AUC of each category. We quantify the model performance by calculating the area under the ROC curve (AUC), and the closer the AUC value is to 1, the better the classification effect of the model. As can be seen from the figure, the AUC values of the OM-VST network are close to 1 for all categories, indicating its robust classification ability when dealing with this complex dataset. In addition, the AUC values of both micro- and macro-averages show that the network achieves a high classification effect on the whole and individual categories, proving that it still performs well in diverse and complex environments.

## 5 Discussion

**Fig 11:**
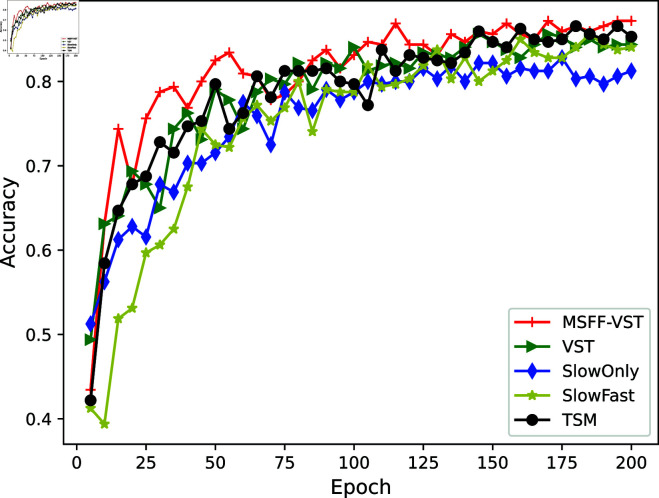
Comparison of model accuracy.

This paper analyzes the OM-VST model experimentally and compares it with the VST model and other state-of-the-art video classification models to validate its performance in video classification tasks. These comparative experiments are conducted under strict control in the same configuration environment to ensure consistency in experimental conditions and to fairly and accurately evaluate the performance of each model. Temporal Shift Module (TSM) is an efficient spatiotemporal modeling method for video understanding [35]. It utilizes channel shift operations in the time dimension to enhance the modeling ability of video features in the time dimension, thereby achieving an efficient understanding of video content without increasing computational complexity. The SlowFast model is a dual-channel model used for video recognition tasks, which processes spatial and temporal information of videos through channels of different speeds. This model includes two key components, the Slow pathway and the Fast pathway, each responsible for capturing static and dynamic information respectively in the video, and the Single Channel SlowOnly model, which only captures static data in the Slow pathway [36]. This paper compares the accuracy, recall, precision, mAP, macro-F1, and micro-F1 metrics of the model with those of these models. Fig 11 displays the comparison of model accuracy.

Below is the Accuracy comparison graph between the OM-VST network and the above networks. As shown in Fig 11, overall, the Accuracy of each model during the network training process is rapidly increasing, and when the training epoch reaches 150, the Accuracy stabilizes. The OM-VST model shows a higher Accuracy of 87.19*%* than the other models. This is followed by the TSM model with an Accuracy of 85.31*%*. Then comes the VST model with an Accuracy of 84.38*%*, and the SlowOnly model with an Accuracy of only 81.25*%*. The OM-VST model shows a 2.81*%* improvement in Accuracy compared to the VST model and is also higher than the other models involved in the experiment. Our proposed two modules can help the network extract more feature information and improve classification performance.

**Fig 12:**
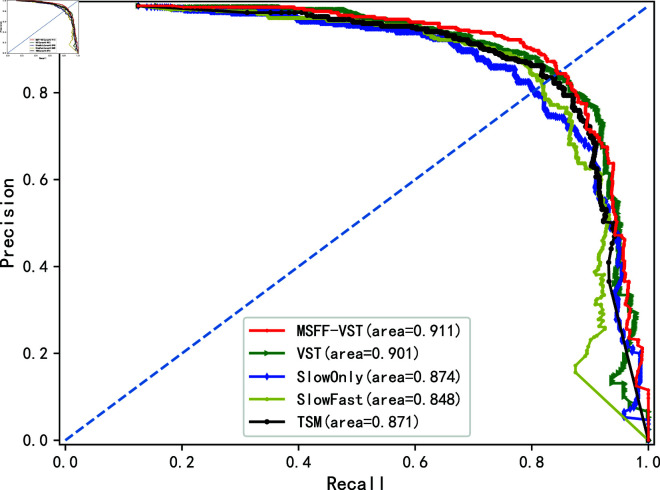
P-R curve.

To further validate the performance of the OM-VST model, we made a comparison plot of the P-R curves of multiple models, as shown in Fig 12. The P-R curve analyzes the relationship between Precision and Recall. We can evaluate the average precision of the model at each recall level based on the area under the P-R curve, i.e., the Average Precision (AP) value. In the figure, the OM-VST model has the highest AP of 0.911, followed by the VST model at 0.901, then SlowOnly at 0.874 and TSM at 0.871, and SlowFast at 0.848. In addition, according to the intersection point of the P-R curve and the diagonal, the equilibrium point of the P-R curve can be derived. The larger the value of the equilibrium point, the better the model’s performance. The equilibrium point of the OM-VST model is higher than that of other models. Combined with the above indicators, it verifies that the OM-VST model has high performance.

Below are the Accuracy, mAP, macro-F1, and micro-F1 values for each evaluation metric of the models participating in the experiment, as shown in Table 3. The OM-VST model performs optimally in all evaluation metrics; for example, the OM-VST model has an accuracy of 87.19*%*, which exceeds the second TSM model by 1.88*%* and improves by 2.81*%* compared to the VST model. 2.81*%*; the mAP value of the OM-VST model is 0.911, exceeding the second VST model by 0.01 and improving by 0.063 compared to the lowest SlowFast model; and the Macro-F1 value of the OM-VST model is 0.871, improving by 0.018 compared to the second TSM model, and improving by 0.03 compared to the VST model; The Micro-F1 value of OM-VST model is 0.872, which improves 0.019 compared to the second TSM model and improves 0.028 compared to the VST model. The OM-VST model performs better than mainstream video classification models such as VST, TSM, etc.

**Table 3:** Performance comparison of different models.

*Model*	Accuracy	mAP	Macro-F1	Micro-F1
*SlowOnly*	81.25%	0.874	0.812	0.813
*SlowFast*	84.06%	0.848	0.845	0.844
*TSM*	85.31%	0.871	0.853	0.853
*VSTdel*	84.38%	0.901	0.841	0.844
*OM*–*VST*	87.19%	0.911	0.871	0.872

In the following, we compare the distribution of the number of Params and the computation of the OM-VST model with the VST model. As shown in Table 4, the table demonstrates the Params and GFLOPs of the two models with the Params of each stage in the model. In the table, the overall parameters of the OM-VST model are reduced by 15.24M or 54.7*%*, and the GFLOPs are reduced by 21.94G, or 31.4*%*, compared with the VST model. These Params reductions are mainly concentrated in Stage 3 and Stage 4 of the model because, in that stage, we remove some unnecessary network layers, which significantly reduces the model’s size. There is a slight increase in Params in Stage 1 and Stage 2 because we do not remove the network layer for that stage but only add multi-scale feature fusion in the feature extraction stage. Overall, we performed network scale compression to make it more compact and reduce the resource loss in network computation.

**Table 4:** Comparison of model parameters.

*Model*	Params	Stage1	Stage2	Stage3	Stage4	GFLOPs
*VST*	27.86	0.298	1.186	11.83	14.176	69.95
*OM *–*VST*	12.62	0.604	2.388	3.549	5.956	48.01

### Ablation experiment

To validate the OM-VST model’s performance, we will perform ablation experiments to evaluate the impact of the optimized downsampling module and the multiscale feature fusion module on the model’s overall performance. Compare the Accuracy, mAP, Macro-F1, and Micro-F1 of the OM-VST model, the M-VST model with the optimized downsampling module removed, the O-VST model with the multiscale feature fusion module removed, and the VST model with both modules removed.

As seen from Table 5, the performance of the OM-VST model decreases after removing either the optimization downsampling module or the multiscale feature fusion module. The O-VST model containing only the optimized downsampling module outperforms the VST model in all evaluation metrics, with a 2.49*%* improvement in Accuracy, 0.006 improvement in mAP, 0.027 improvement in Macro-F1, and 0.025 improvement in Macro-F1. The M-VST model, which contains only the multiscale feature fusion module, also improves several evaluation metrics. Accuracy was enhanced by 0.62*%*, Macro-F1 by 0.027, and Micro-F1 by 0.025. The model’s performance gradually decreases when the optimized downsampling module and the multiscale feature fusion module are removed. This suggests that these two modules play a role in the model’s performance improvement.

**Table 5:** Comparison of ablation experiment results.

*Methods*	Accuracy 1	mAP1	Macro-F1	Micro-F1
*VST*	84.38%	0.901	0.841	0.844
*M*–*VST*	85.00%	0.890	0.851	0.850
*O*–*VST*	86.87%	0.907	0.868	0.869
*OM*–*VST*	87.19%	0.911	0.871	0.872

## 6 Conclusion

We study the video classification problem and propose an optimized downsampling module combined with multiscale feature fusion for the video classification model, i.e., the OM-VST model. Specifically, to solve the problem of insufficient perception of feature information in downsampling by traditional methods, we adopt the idea of large kernel convolution in the downsampling module and use the Inverted Bottleneck architecture to increase the feature information perception field and reduce the loss of information in the training process. On this basis, we incorporate a multiscale feature fusion module in the feature extraction stage, which captures richer local features while maintaining the complete morphology of the object in the feature map by weighting the feature information from two different convolutional branches. We verify the performance of the OM-VST model through comparison experiments with other models. According to several evaluation metrics, the OM-VST model performs better than mainstream video classification models, with a 2.81*%* improvement in accuracy and a 54.7*%* reduction in the number of parameters.

The OM-VST model also has some limitations. Because we have lightened the model structure more, the convergence speed and fitting ability of the model in large datasets is insufficient. If the computational resources are sufficient, subsequent studies will be explored using large-scale datasets and the SOTA model for specific tasks. The feature fusion method used in the feature fusion stage is the weight summing feature fusion method; in the subsequent research, we will introduce the attention mechanism for feature fusion; the basic method I3D used in the classification task header, we intend to replace it with other model classification headers, such as X3D, Slowfast, etc., and carry out further optimization.

## Supporting information

S1 Text Code for key modules. (PDF)
