# Supplementary material for: OM-VST: A video action recognition model based on optimized downsampling module combined with multi-scale feature fusion
Source: PLoS One. 2025 Mar 6;20(3):e0318884. doi: 10.1371/journal.pone.0318884 (PMC11884693; doi:10.1371/journal.pone.0318884)
Supplement: S1 Text — (PDF) [file pone.0318884.s001.pdf]

Supplementary Algorithms

Algorithm 1: our proposed algorithm. OM-Video Swin Transformer (OM-VST) Model

Algorithm 1 OM-Video Swin Transformer (OM-VST) Model

Input: Video data with dimensions $B \times C \times T \times H \times W$
Output: Predicted features y with dimensions $B \times 8$
Step 1: Preprocessing
$P \leftarrow 3D\_Patch\_Partition(V)$ {Divide video into 3D patches, output $B \times T \times H \times W$}
$E \leftarrow Linear\_Embedding(P)$ {Generate initial feature embeddings, output $B \times T \times H \times W \times C$}
Step 2: Feature Extraction
$F_1 \leftarrow Video\_Swin\_Transformer\_Block(E)$ {Stage 1: Extract features, output $B \times \frac{T}{2} \times \frac{H}{2} \times \frac{W}{2} \times 2C$}
$F_1^{down} \leftarrow Downsample(F_1)$ {Downsample Stage 1, output $B \times \frac{T}{2} \times \frac{H}{2} \times \frac{W}{2} \times 2C$}
$F_2 \leftarrow Video\_Swin\_Transformer\_Block(F_1^{down})$ {Stage 2, output $B \times \frac{T}{2} \times \frac{H}{4} \times \frac{W}{4} \times 4C$}
$F_2^{down} \leftarrow Downsample(F_2)$ {Downsample Stage 2, output $B \times \frac{T}{2} \times \frac{H}{4} \times \frac{W}{4} \times 4C$}
$F_3 \leftarrow Video\_Swin\_Transformer\_Block(F_2^{down})$ {Stage 3, output $B \times \frac{T}{2} \times \frac{H}{8} \times \frac{W}{8} \times 8C$}
$F_3^{down} \leftarrow Downsample(F_3)$ {Downsample Stage 3, output $B \times \frac{T}{2} \times \frac{H}{8} \times \frac{W}{8} \times 8C$}
$F_4 \leftarrow MS\_Convolution(F_3^{down})$ {Stage 4 with Multi-Scale Convolution, output $B \times \frac{T}{2} \times \frac{H}{16} \times \frac{W}{16} \times 16C$}
$F_4^{down} \leftarrow Downsample(F_4)$ {Downsample Stage 4, output $B \times \frac{T}{2} \times \frac{H}{16} \times \frac{W}{16} \times 16C$}
Step 3: Classification
$F_{pool} \leftarrow AvgPool(F_4^{down})$ {Apply global average pooling}
$F_{drop} \leftarrow Dropout(F_{pool})$ {Apply dropout for regularization }
$y \leftarrow Linear\_Classification(F_{drop})$ {Final classification layer, output $B \times 8$}
return y

Algorithm 2: our proposed algorithm. optimized downsampling module

Algorithm 2 optimized downsampling module

Input: Tensor x with dimensions $B \times T \times H \times W \times C$
Output: Processed tensor y with dimensions $B \times T \times H/2 \times W/2 \times 2C$
Parse tensor dimensions: $(B, T, H, W, C) \leftarrow \text{shape}(x)$ {Extract batch size, time, height, width, and channels from the input tensor}
if $H\%2 = 1$ **or** $W\%2 = 1$ **then**
  Pad x to make height and width even {Ensure the height and width are even to support spatial operations}
end if
Step 1: Split and Concatenate Sub-Tensors
Split x into four sub-tensors: x_0, x_1, x_2, x_3
Concatenate: $x \leftarrow \text{concat}(x_0, x_1, x_2, x_3, \text{dim} = -1)$ {Combine the 4 sub-tensors along the channel dimension to get $B \times T \times H/2 \times W/2 \times 4C$}
Normalize: $x \leftarrow \text{norm}(x)$ {Apply layer normalization (initialized as norm_layer($4 \cdot$ dim)) across the tensor}
Apply linear reduction: $x \leftarrow \text{reduction}(x)$ {Reduce the channel dimension from $4C$ to $2C$ using a linear layer (initialized as $\text{Linear}(4 \cdot \text{dim}, 2 \cdot \text{dim})$)}
Step 2: Depthwise Convolution
Apply depthwise convolution: $x \leftarrow \text{dwconv}(x)$ {Apply depthwise convolution to process each channel independently (initialized as Conv2d with groups=$2C$)}
Step 3: Pointwise Convolutions and Activation
Apply first pointwise convolution: $x \leftarrow \text{pwconv1}(x)$ {Increase channel dimension from $2C$ to $8C$ (initialized as $\text{Linear}(\text{dim} \times 2, \text{dim} \times 8)$)}
Apply GELU activation: $x \leftarrow \text{act}(x)$ {Apply GELU activation function for non-linearity (initialized as GELU)}
Apply second pointwise convolution: $x \leftarrow \text{pwconv2}(x)$ {Reduce channel dimension from $8C$ back to $2C$ (initialized as $\text{Linear}(\text{dim} \times 8, \text{dim} \times 2)$)}
Step 4: Apply Scaling (if enabled)
if $\gamma \neq 0$ **then**
  Scale the output: $x \leftarrow \gamma \times x$ {Apply scaling factor γ (initialized as $1e^{-6}$) if non-zero}
end if
Step 5: Residual Connection
Add residual connection: $x \leftarrow \text{shortcut} + \text{drop\_path}(x)$ {Add the input tensor *shortcut* to the output, with stochastic depth applied (initialized with drop_rate)}
Step 6: Final Normalization and Output
Normalize the final output: $x \leftarrow \text{norm1}(x)$ {Apply final normalization (initialized as $\text{LayerNorm}(\text{dim} \times 2)$)}
Assign output: $y \leftarrow x$ {Assign the processed tensor to the output variable}
return y {Return the final processed tensor}

Algorithm 3: our proposed algorithm. multi-scale feature fusion module

Algorithm 3 our proposed algorithm.multi-scale feature fusion module

Input: Tensor x with dimensions $B \times C \times H \times W$
Output: Processed tensor y with dimensions $B \times C \times H \times W$
Parse tensor dimensions: $(B, C, H, W) \leftarrow \text{shape}(x)$
Get weight:
    weight $\leftarrow$ get_weight(x)
    $\text{h}, \text{w} \leftarrow$ weight.shape[2:] {Extract height and width of the weight tensor.}
Normalize weights:
    weighted $\leftarrow$ weight.view(B, C, kernel_size^2, h, w).$softmax(2)$ {Apply softmax along the kernel dimension to normalize the weights.}
Generate features:
    feature $\leftarrow$ generate_feature(x).$view(B, C$, kernel_size^2, $h, w)$ {Generate feature map using depthwise convolution and reshape for element-wise operations.}
Multiply feature with weights:
    weighted_data $\leftarrow$ feature $\times$ weighted {Element-wise multiplication of features and normalized weights.}
Rearrange data:
    conv_data $\leftarrow$ rearrange(weighted_data,
        'b c (n1 n2) h w -> b c (h n1) (w n2)',
        $n1$ = kernel_size, $n2$ = kernel_size)
Final convolution:
    y $\leftarrow$ conv($conv\_data$) {Apply final convolution and return the output.}
return y
